# Interleukin-18 Mediates Immune Responses to *Campylobacter jejuni* Infection in Gnotobiotic Mice

**DOI:** 10.1371/journal.pone.0158020

**Published:** 2016-06-20

**Authors:** Stefan Bereswill, Marie E. Alutis, Ursula Grundmann, André Fischer, Ulf B. Göbel, Markus M. Heimesaat

**Affiliations:** Department of Microbiology and Hygiene, Charité - University Medicine Berlin, Berlin, Germany; The Ohio State University, UNITED STATES

## Abstract

**Background:**

Human *Campylobacter jejuni* infections are progressively rising worldwide. Information about the molecular mechanisms underlying campylobacteriosis, however, are limited. In the present study we investigated whether cytokines such as IL-23, IL-22 and IL-18, which share pivotal functions in host immunity, were involved in mediating intestinal and systemic immunopathological responses upon *C*. *jejuni* infection.

**Methodology/Principal Findings:**

To assure stable infection, gnotobiotic (i.e. secondary abiotic) IL-23p19^-/-^, IL-22^-/-^ and IL-18^-/-^ mice were generated by broad-spectrum antibiotic treatment. Following peroral *C*. *jejuni* strain 81–176 infection, mice of all genotypes harbored comparably high pathogenic loads in their intestines. As compared to wildtype controls, however, IL-18^-/-^ mice displayed less distinct *C*. *jejuni* induced sequelae as indicated by less pronounced large intestinal shrinkage and lower numbers of apoptotic cells in the colonic epithelial layer at day 8 postinfection (p.i.). Furthermore, lower colonic numbers of adaptive immune cells including regulatory T cells and B lymphocytes were accompanied by less distinct secretion of pro-inflammatory cytokines such as TNF and IFN-γ and lower IL-17A mRNA expression levels in colonic *ex vivo* biopsies of infected IL-18^-/-^ as compared to wildtype mice. Upon *C*. *jejuni* infection, colonic IL-23p19 expression was up-regulated in IL-18^-/-^ mice only, whereas IL-22 mRNA levels were lower in uninfected and infected IL-23p19^-/-^ as well as infected IL-18^-/-^ as compared to respective wildtype control mice. Remarkably, not only intestinal, but also systemic infection-induced immune responses were less pronounced in IL-18^-/-^ mice as indicated by lower TNF, IFN-γ and IL-6 serum levels as compared to wildtype mice.

**Conclusion/Significance:**

We here show for the first time that IL-18 is essentially involved in mediating *C*. *jejuni* infection in the gnotobiotic mouse model. Future studies need to further unravel the underlying regulatory mechanisms orchestrating pathogen-host interaction.

## Introduction

During the past decade, human infections with the zoonotic pathogen *Campylobacter jejuni* have progressively risen in developed as well as developing countries [1–3]. Whereas *C*. *jejuni* is considered a commensal strain in the intestinal tract of many wild and domestic animal species, it can cause human disease of considerable variability following transmission via the food chain [4–6]. Whereas infected individuals may be asymptomatic or exhibit rather mild symptoms including watery diarrhea, other patients suffer from acute ulcerative enterocolitis with inflammatory bloody diarrhea and abdominal cramps lasting for up to a few days or even weeks [7]. In the vast majority of cases, the course of disease is self-limited. On rare occasions, however, post-infectious sequelae such as reactive arthritis or neurological complications including Guillain-Barré and Miller-Fisher syndromes may arise with a latency of several weeks to months postinfection (p.i.) [7, 8]. Intestinal immunopathology in human campylobacteriosis is characterized by histological changes including apoptosis, crypt abscesses, ulcerations and pronounced influx of distinct pro-inflammatory immune cell subsets including lymphocytes and neutrophils into the intestinal mucosa and lamina propria [9, 10]. Despite its global importance, our understanding of the molecular mechanisms underlying campylobacteriosis, however, is limited due to fundamental shortcomings in experimental infection model systems. Chicken, primates, newborn piglets, weanling ferrets, and gnotobiotic canine pups have been applied for studying pathogen-host interactions with limited success only, whereas mice are prevented from stable *C*. *jejuni* colonization due to the physiological colonization resistance exerted by their conventional intestinal microbiota [3, 7]. We have recently shown that colonization resistance can be abrogated by depletion of the commensal intestinal microbiota following broad-spectrum antibiotic treatment [11]. In turn, generated gnotobiotic (i.e. secondary abiotic) mice could be stably colonized by the pathogen at high loads upon peroral challenge and displayed distinct pathogen-induced pro-inflammatory immune and apoptotic responses in their intestinal tract, hence mimicking key features of campylobacteriosis in men [11]. Thus, the gnotobiotic mice infection model has been proven suitable to further dissect *C*. *jejuni*-host interactions.

Our previous studies revealed that IL-23p19, IL-22 and IL-18, which share pivotal functions in host immunity [12–15], were upregulated in the large intestines of both conventional infant [16] and secondary abiotic adult mice following peroral *C*. *jejuni* infection [17]. Recently, IL-23 was highlighted as a master regulator of mucosal immune responses upon intestinal infection and inflammation [18], whereas IL-22 belonging to the IL-10 family exerts potent antimicrobial and tissue-protective, but also pro-inflammatory properties [19, 20]. Particularly in the intestinal tract, IL-22 acts in a dichotomous fashion depending on the respective compartment. In the large intestines, for instance, IL-22 exerts its anti-inflammatory properties [20]. Our group showed recently that in the small intestinal tract, however, IL-22 acts as a pro-inflammatory cytokine, given that acute *Toxoplasma gondii* induced ileitis was caused by IL-23p19 dependent IL-22 induction [21–23].

IL-18 was initially identified as a cytokine promoting T helper cell (Th) -1 development and IFN-γ production [14]. For generating the active form of IL-18, inflammasome activation and caspase-mediated enzymatic cleavage is required [24]. Our recent studies revealed that IL-18 mRNA expression in intestinal epithelial cells is induced by IL-22 following *T*. *gondii* infection, whereas, IL-18 amplified IL-22 production from innate lymphoid cells (ILCs) and Th-1 mediated intestinal inflammation [23]. So far, however, it is unclear whether such a mutual regulation between IL-22 and IL-18 also holds true for *C*. *jejuni* infection. Overall, information about the distinct roles of cytokines belonging to the IL-23 / IL-22 / IL-18 axis in *C*. *jejuni* infection are scarce.

In the present study we hence aimed to elucidate the impact of the IL-23 / IL-22 / IL-18 axis in *C*. *jejuni*-host interaction and investigated macroscopic and microscopic pathogen-induced sequelae as well as local and systemic pro-inflammatory immune responses in perorally *C*. *jejuni* infected gnotobiotic adult mice that were gene-deficient for either IL-23p19, IL-22 or IL-18. For the first time we were able to show that IL-18 is essentially involved in mediating intestinal and systemic immune responses upon murine *C*. *jejuni* infection.

## Methods

### Ethics statement

All animal experiments were conducted according to the European Guidelines for animal welfare (2010/63/EU) with approval of the commission for animal experiments (ethical committee) headed by the “Landesamt für Gesundheit und Soziales” (LaGeSo, Berlin, registration number G0135/10). Animal welfare was monitored twice daily by assessment of body weights and clinical conditions including occurrence of blood in murine fecal samples. Suffering of mice was ameliorated whenever applicable according to the legacy of the animal ethical committee. At day of necropsy (i.e. day 8 p.i.) mice were anaethesized by isofluran (Abbott, Greifswald, Germany) inhalation.

### Mice and *C*. *jejuni* infection

Female IL-23p19^-/-^, IL-22^-/-^ and IL-18^-/-^ mice (all in C57BL/6j background) as well as age- and sex-matched C57BL/6j wildtype (WT) control mice were bred and maintained within the same specific pathogen free (SPF) unit in the Forschungseinrichtungen für Experimentelle Medizin (FEM), Charité—University Medicine Berlin. In order to confirm absence of IL-23p19, IL-22 or IL-18 gene expression, genomic DNA was isolated and disruption of either gene confirmed by polymerase chain reaction (PCR) [21]. Gnotobiotic (i.e. secondary abiotic) mice with a virtually depleted gastrointestinal microbiota were generated by broad-spectrum antibiotic treatment as described earlier [25]. In brief, mice were transferred to sterile cages and treated by adding ampicillin/sulbactam (1 g/L; Pfizer, Berlin, Germany), vancomycin (500 mg/L; Hexal, Holzkirchen, Germany), ciprofloxacin (200 mg/L; Hexal), imipenem (250 mg/L; Fresenius Kabi, Graz, Austria), and metronidazole (1 g/L; Braun, Melsungen, Germany) to the drinking water *ad libitum* starting eight weeks of age and continued for 10 weeks before the infection experiment. Three days before infection, the antibiotic cocktail was replaced by sterile tap water (*ad libitum*). On two consecutive days (day 0 and day 1) mice were perorally infected with 10^9^ colony forming units (CFU) of viable *C*. *jejuni* strain 81–176 in a volume of 0.3 mL phosphate buffered saline (PBS) by gavage as described earlier [11]. Mice were continuously kept in a sterile environment (autoclaved food and tap water) and handeled under strict aseptic conditions. Uninfected gnotobiotic WT, IL-23p19^-/-^, IL-22^-/-^ and IL-18^-/-^ mice served as respective negative (uninfected) control groups. A minimum of three mice per group were included into the experiments.

### Sampling procedures

Mice were sacrificed at day 8 postinfection (p.i.) by isofluran treatment (Abbott, Greifswald, Germany). Cardiac blood and tissue samples from colon, mesenteric lymph nodes (MLN), spleen, liver and kidney were asserved under sterile conditions. Absolute large intestinal lengths were determined by measuring the distance from the ascending colon leaving the caecum to the rectum by a ruler. Colonic *ex vivo* biopsies were collected in parallel for immunohistochemical, microbiological and immunological analyses. Immunohistopathological changes were assessed in sections (5 μm) of colonic samples that were immediately fixed in 5% formalin and embedded in paraffin.

### Immunohistochemistry

*In situ* immunohistochemical analysis of colonic paraffin sections was performed as described previously [17, 26]. Primary antibodies against cleaved caspase-3 (Asp175, Cell Signaling, Beverly, MA, USA, 1:200), Ki67 (TEC3, Dako, Denmark, 1:100), CD3 (#N1580, Dako, 1:10), FOXP3 (FJK-16s, eBioscience, 1:100), B220 (eBioscience, 1:200), and F4/80 (# 14–4801, clone BM8, eBioscience, San Diego, CA, USA, 1:50) were used. For each animal, the average number of positively stained cells within at least six high power fields (HPF, 0.287 mm^2^, 400 x magnification) were determined microscopically by a double-blinded investigator.

### Quantitative analysis of bacterial colonization and translocation

Viable *C*. *jejuni* were detected in feces over time p.i. or in luminal colonic samples at time of necropsy (i.e. day 8 p.i.), dissolved in sterile PBS and serial dilutions cultured on Karmali- and Columbia-Agar supplemented with 5% sheep blood (Oxoid) for two days at 37°C under microaerobic conditions using CampyGen gas packs (Oxoid). To quantify bacterial translocation, *ex vivo* biopsies derived from MLNs, spleen, liver and kidney were homogenized in 1 mL sterile PBS, whereas cardiac blood (≈100 μL) was directly streaked onto Karmali-Agar and Columbia-Agar supplemented with 5% sheep blood and cultivated accordingly. The respective weights of fecal or tissue samples were determined by the difference of the sample weights before and after asservation. The detection limit of viable pathogens was ≈100 colony forming units (CFU) per gram (g) as assessed by direct plating.

### Cytokine detection in supernatants of colonic *ex vivo* biopsies

Colonic tissue samples were cut longitudinally and washed in PBS. Strips of approximately 1 cm^2^ intestinal tissue were placed in 24-flat-bottom well culture plates (Nunc, Wiesbaden, Germany) containing 500 μL serum-free RPMI 1640 medium (Gibco, life technologies, Paisley, UK) supplemented with penicillin (100 U/mL) and streptomycin (100 μg/mL; PAA Laboratories). After 18 h at 37°C, culture supernatants or serum samples were tested for TNF, IFN-γ, and IL-6 by the Mouse Inflammation Cytometric Bead Assay (CBA; BD Biosciences) on a BD FACSCanto II flow cytometer (BD Biosciences).

### Real-time PCR

RNA was isolated from snap frozen colonic *ex vivo* biopsies, reverse transcribed and analyzed as described previously [21]. In brief, murine IL-23p19, IL-22, IL-18, IL-17A and IL-1β mRNA expressions were detected and analyzed using Light Cycler Data Analysis Software (Roche). The mRNA of the housekeeping gene for hypoxanthine-phosphoribosyltransferase (HPRT) was used as reference, the mRNA expression levels of the individual genes were normalized to the lowest measured value and expressed as fold expression (Arbitrary Units).

### Statistical analysis

Medians, means and levels of significance were determined using Mann-Whitney U test (GraphPad Prism v6.05, La Jolla, CA, USA) as indicated. Two-sided probability (*P*) values ≤ 0.05 were considered significant. Experiments were reproduced at least twice.

## Results

### Colonization properties of *C*. *jejuni* in gnotobiotic mice lacking IL-23p19, IL-22 or IL18

**Fig 1:**
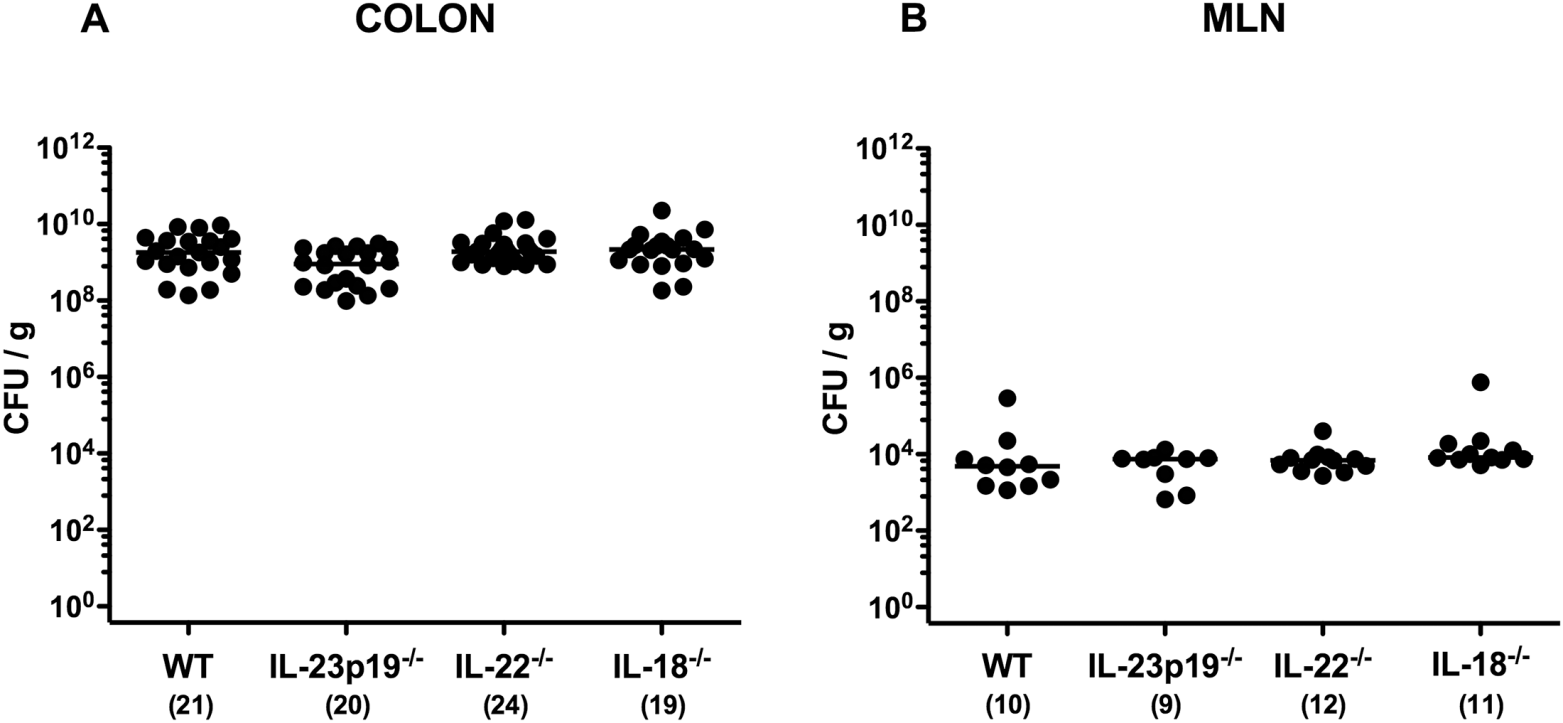
Intestinal *C*. *jejuni* loads in perorally infected gnotobiotic mice lacking IL-23p19, IL-22 or IL-18. Gnotobiotic wildtype (WT), IL-23p19^-/-^, IL-22^-/-^ and IL-18^-/-^ mice were generated by broad-spectrum antibiotic treatment and perorally infected with *C*. *jejuni* strain 81–176 by gavage at day 0 and day 1. Pathogenic loads (colony forming units (CFU) per gram) were determined in **(A)** colonic luminal samples and **(B)** homogenates of mesenteric lymphnodes (MLN) at day 8 postinfection by culture. Numbers of analyzed mice (in parentheses), medians (black bars) and level of significance (p-value) determined by Mann-Whitney U test are indicated. Data were pooled from four independent experiments.

In the present study we dissected the role of cytokines belonging to the IL-23 / IL-22 / IL-18 axis in murine campylobacteriosis. To address this, gnotobiotic IL-23p19^-/-^, IL-22^-/-^, IL-18^-/-^ and corresponding WT control mice with a virtually depleted commensal intestinal microbiota were generated by broad-spectrum antibiotic treatment [11, 25–29]. Upon peroral infection with 10^9^ CFU of *C*. *jejuni* strain 81–176 on two consecutive days (day 0 and day 1), mice of all genotypes were stably infected with comparably high pathogenic loads of approximately 10^9^ CFU per g luminal colonic sample at day 8 p.i. (Fig 1A). At the same time point, viable *C*. *jejuni* could also be isolated from MLNs of gnotobiotic mice, irrespective of their genotype, with median loads of 10^4^ CFU per g organ homogenate (Fig 1B).

### Macroscopic and microscopic sequelae of *C*. *jejuni* infection in gnotobiotic mice lacking IL-23p19, IL-22 or IL18

We next surveyed the effect of IL-23, IL-22 and IL-18 in mediating inflammatory responses during murine *C*. *jejuni* infection. Overall, mice of all genotypes were rather uncompromized from their clinical aspect, given that only in single cases (i.e. 9.5%, 5.0%, 8.3% and 10.5%, respectively) occult blood could be detected microscopically in fecal samples derived from WT, IL-23^-/-^, IL-22^-/-^ and IL-18^-/-^ mice at day 8 p.i. (S1 Fig). As early as 24 hours following the first of two consecutive *C*. *jejuni* infections, however, more than 20% of IL-18^-/-^ mice as compared to 5% of IL-23p19^-/-^, but none of IL-22^-/-^ and WT mice displayed fecal blood (S1A Fig). Whereas later during the course of infection IL-18^-/-^ mice had recovered and were free of blood in their feces, almost half of WT mice were fecal blood-positive at day 5 p.i. (S1B Fig).

**Fig 2:**
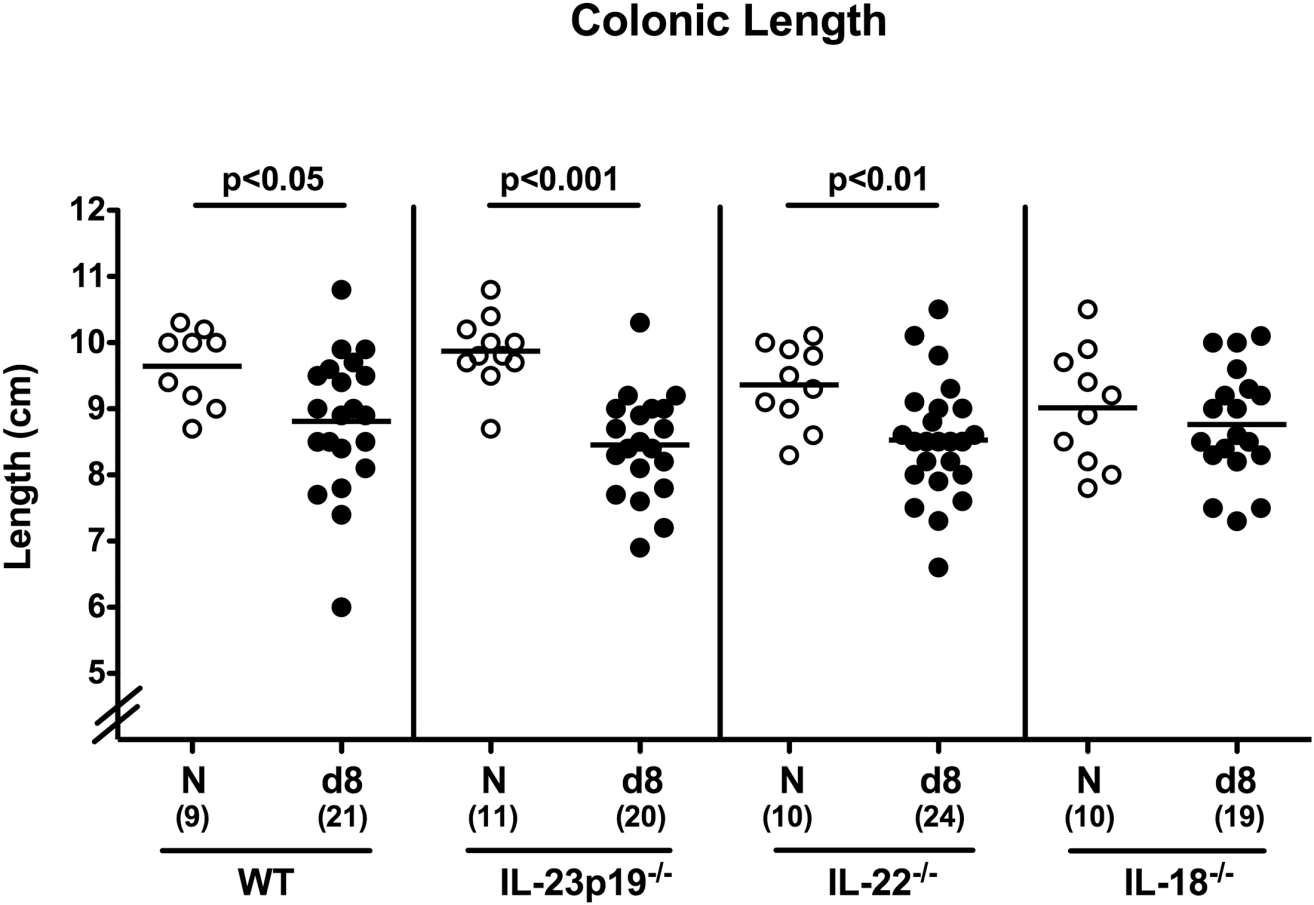
Colonic lengths of perorally *C*. *jejuni* strain 81–176 infected gnotobiotic mice lacking IL-23p19, IL-22 or IL-18. Absolute colonic lengths of mice were determined at day of necropsy (day (d) 8 postinfection). Naive (N) mice served as uninfected controls. Numbers of analyzed animals (in parentheses), means (black bars) and level of significance (p-value) determined by Mann-Whitney U test are indicated. Data were pooled from four independent experiments.

Since intestinal inflammation results in significant shortening of the intestinal tract [25, 27], we next measured colonic lengths at days of necropsy. In fact, shorter colonic lengths could be observed in WT, IL-23p19^-/-^, and IL-22^-/-^, but, interestingly, not in IL-18^-/-^ mice at day 8 p.i. as compared to respective uninfected control animals (Fig 2), hence pointing towards less distinct *C*. *jejuni* induced inflammatory responses in IL-18^-/-^ mice.

**Fig 3:**
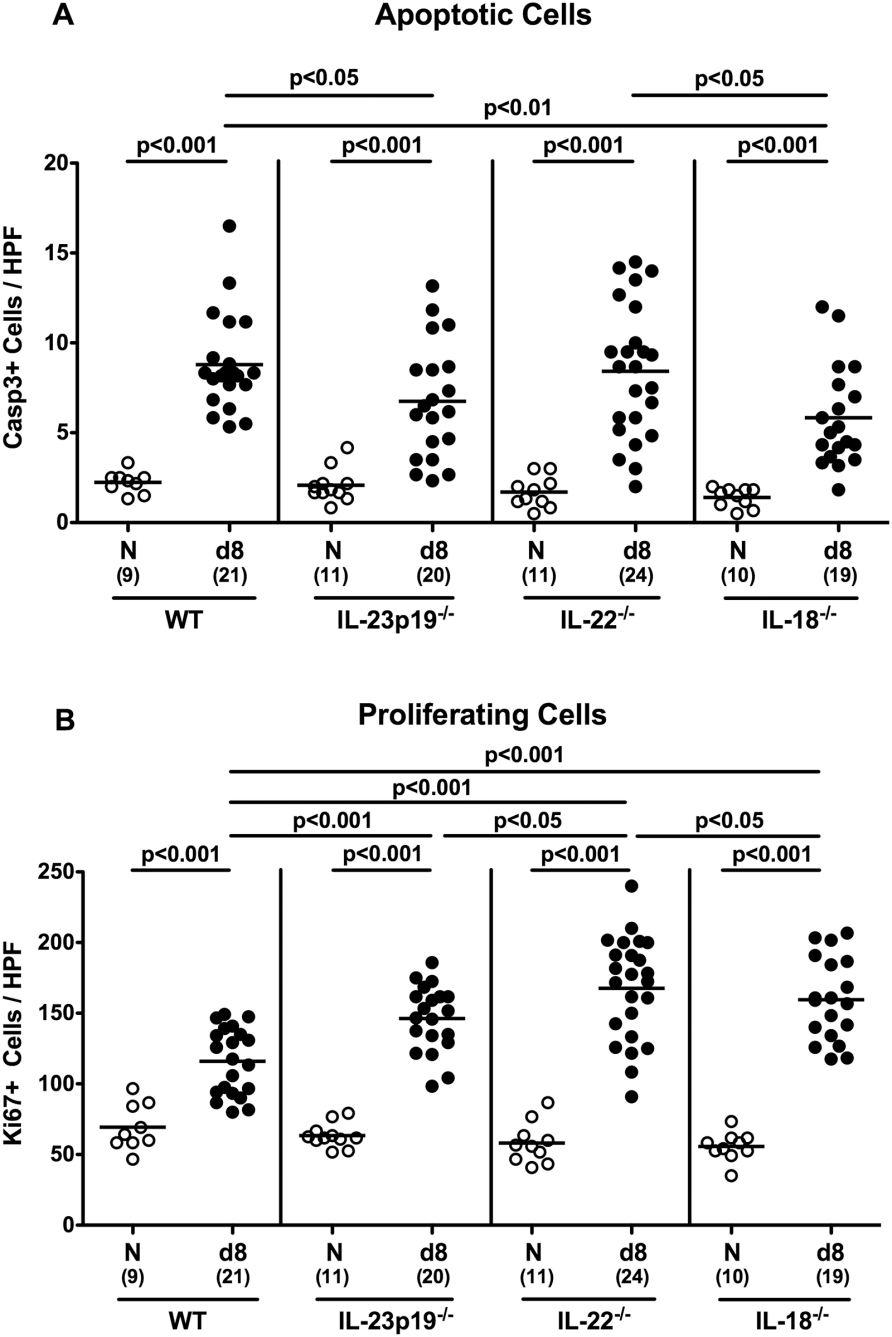
Apoptotic and proliferating cells in the colonic epithelium of *C*. *jejuni* strain 81–176 infected gnotobiotic mice lacking IL-23p19, IL-22 or IL-18. The average number of colonic **(A)** apoptotic cells (positive for caspase-3, Casp3) and **(B)** proliferating cells (positive for Ki67) from at least six high power fields (HPF, 400x magnification) per mouse was determined microscopically in immunohistochemically stained colonic paraffin sections at day (d) 8 (black circles) postinfection. Naive (N) mice served as uninfected controls (white circles). Medians (black bars), level of significance (p-value) determined by Mann-Whitney U test, and numbers of analyzed animals (in parentheses) are indicated. Data were pooled from four independent experiments.

Given that apoptosis is used as an established diagnostic marker in the histopathological evaluation and grading of intestinal disease and a hallmark of murine campylobacteriosis [27], we next determined numbers of caspase-3+ cells within the colonic epithelial layer of infected mice. Irrespective of their genotype, numbers of apoptotic cells increased upon *C*. *jejuni* infection, but less distinctly in IL-18^-/-^ and IL-23p19^-/-^ as compared to WT mice (p<0.05; Fig 3A). We further stained colonic paraffin sections against Ki67 in order to determine proliferative measures of the colonic epithelium counteracting apoptosis following *C*. *jejuni* infection, given that Ki67 comprizes a nuclear protein associated with and necessary for cellular proliferation [30]. Contrary to apoptotic cells, numbers of Ki67 cells were higher in *C*. *jejuni* infected WT and gene-deficient as compared to respective uninfected control mice (p<0.001; Fig 3B). Gene-deficient mice, however, displayed approximately more than 30% higher proliferating colonic mucosal cell numbers as compared to WT controls at day 8 p.i. (p<0.001; Fig 3B). Taken together, the better macroscopic aspects observed in *C*. *jejuni* infected IL-18^-/-^ mice was accompanied by less distinct histological sequelae within the large intestinal mucosa.

### Immune cell responses in *C*. *jejuni* strain 81–176 infected gnotobiotic mice lacking IL-23p19, IL-22 or IL-18

**Fig 4:**
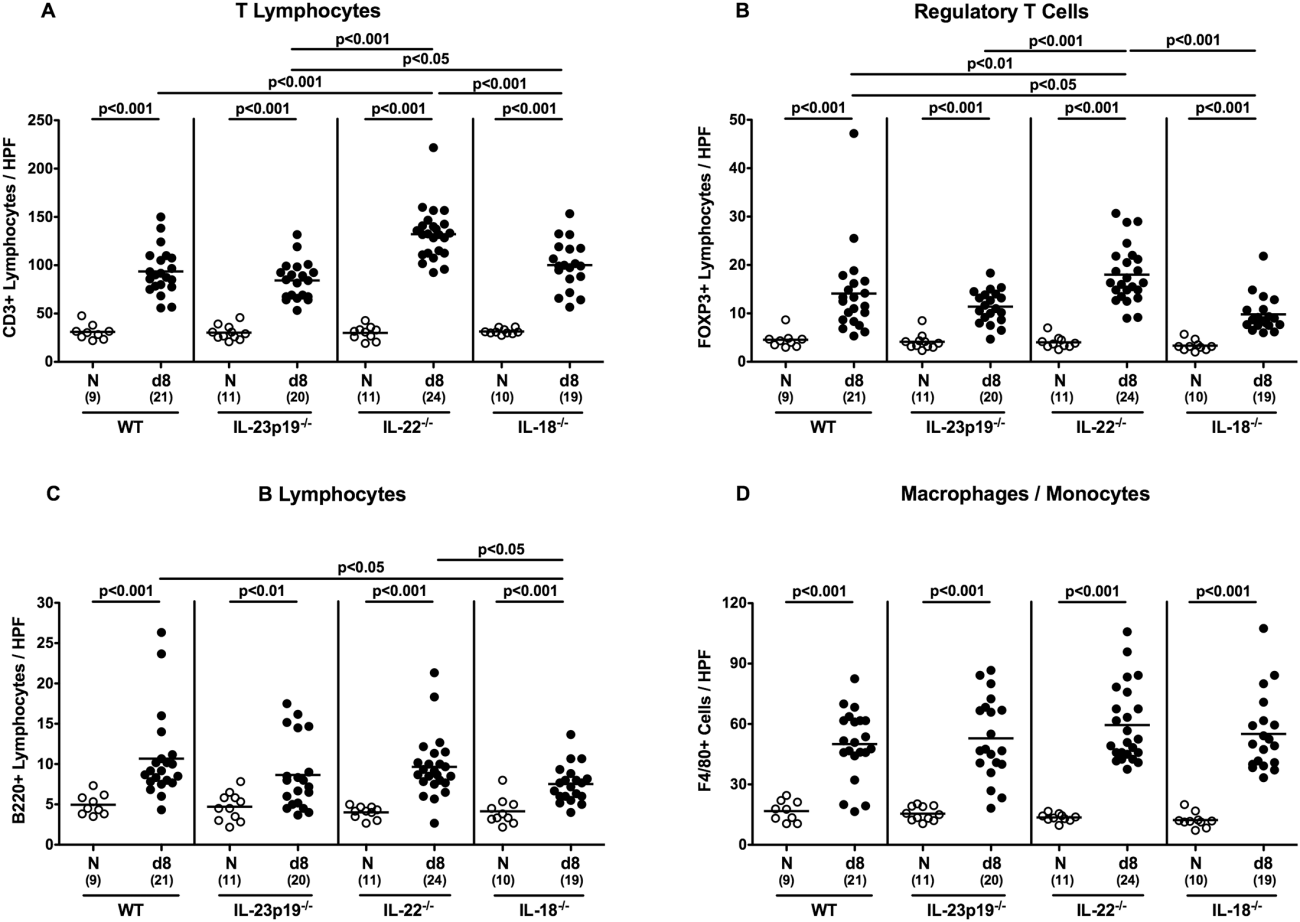
Colonic immune cell responses in *C*. *jejuni* strain 81–176 infected gnotobiotic mice lacking IL-23p19, IL-22 or IL-18. The average number of colonic epithelial cells positive for **(A)** CD3 (T Lymphocytes), **(B)** FOXP3 (Regulatory T Cells, Tregs), **(C)** B220 (B Lymphocytes), and **(D)** F4/80 (Macrophages / Monocytes) from at least six high power fields (HPF, 400x magnification) per animal was determined microscopically in immunohistochemically stained colonic paraffin sections at day (d) 8 (black circles) postinfection. Naive (N) mice served as uninfected controls (white circles). Medians (black bars), level of significance (p-value) determined by Mann-Whitney U test, and numbers of analyzed animals (in parentheses) are indicated. Data were pooled from four independent experiments.

Recruitment of pro-inflammatory immune cells to sites of inflammation is a well-known hallmark of intestinal pathogenic infection including campylobacteriosis [11]. We therefore quantitatively assessed the numbers of innate and adaptive immune cells within the large intestinal mucosa and lamina propria upon *C*. *jejuni* infection by *in situ* immunohistochemical staining of colonic paraffin sections. Irrespective of the genotype of mice, numbers of colonic CD3+ T lymphocytes, FOXP3+ regulatory T cells (Tregs), B220+ B lymphocytes, as well as of F4/80+ macrophages and monocytes increased until day 8 p.i. (p<0.001; Fig 4). In IL-22^-/-^ mice, numbers of colonic T lymphocytes and Tregs were even higher when compared to infected WT mice at day 8 p.i. (p<0.001 and p<0.01, respectively; Fig 4A and 4B), whereas *C*. *jejuni* infected IL-18^-/-^ mice displayed lower Treg and B lymphocyte numbers in their colon as compared to infected WT animals (p<0.05; Fig 4B and 4C). Hence IL-18 mediates adaptive immune responses in *C*. *jejuni* infected gnotobiotic mice.

### Pro-inflammatory cytokine expression in the colon of *C*. *jejuni* strain 81–176 infected gnotobiotic mice lacking IL-23p19, IL-22 or IL-18

**Fig 5:**
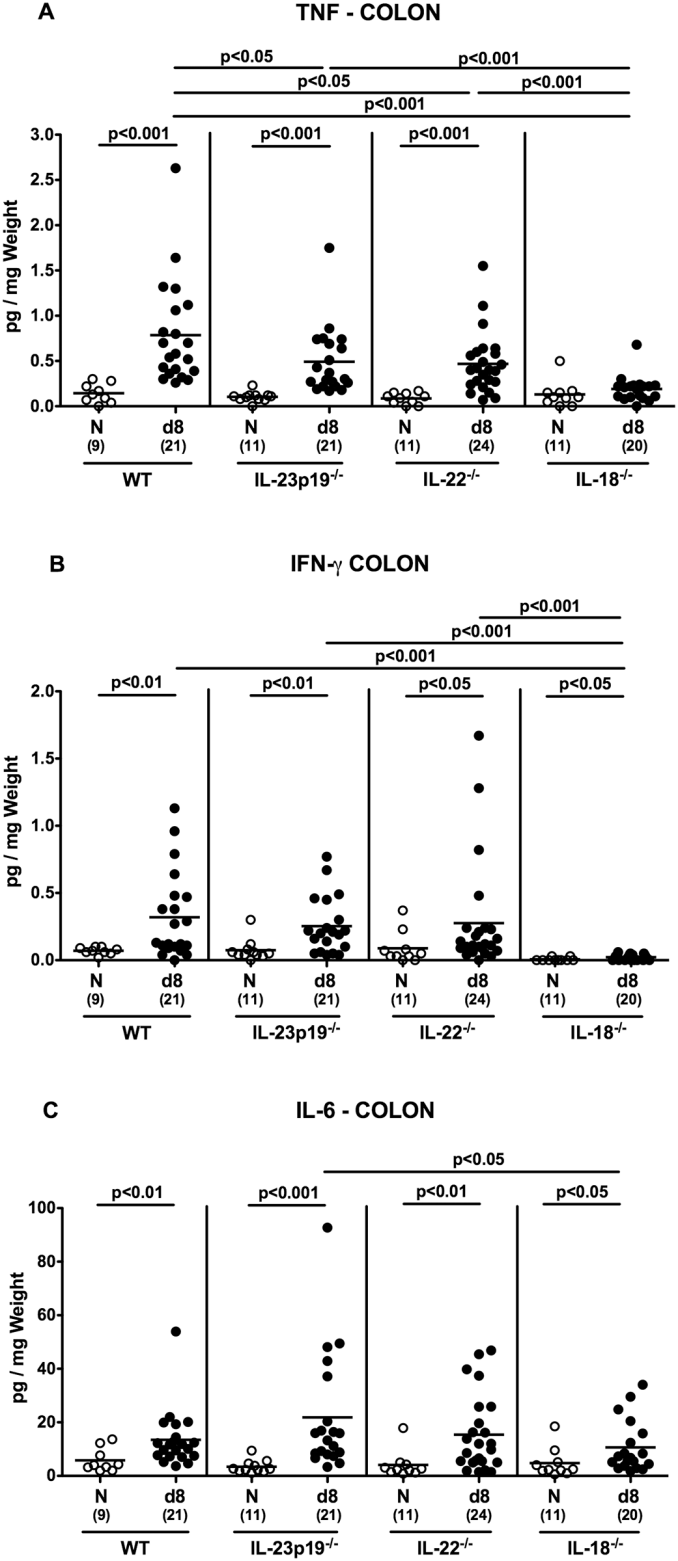
Pro-inflammatory cytokine secretion in colonic *ex vivo* biopsies derived from *C*. *jejuni* strain 81–176 infected gnotobiotic mice lacking IL-23p19, IL-22 or IL-18. **(A)** TNF, **(B)** IFN-γ, and **(C)** IL-6 concentrations were determined in supernatants of colonic *ex vivo* biopsies derived from gnotobiotic wildtype (WT), IL-23p19^-/-^, IL-22^-/-^ and IL-18^-/-^ mice at day (d) 8 (black circles) postinfection. Naive (N) mice served as uninfected controls (white circles). Medians (black bars), level of significance (p-value) determined by Mann-Whitney U test, and numbers of analyzed animals (in parentheses) are indicated. Data were pooled from four independent experiments.

**Fig 6:**
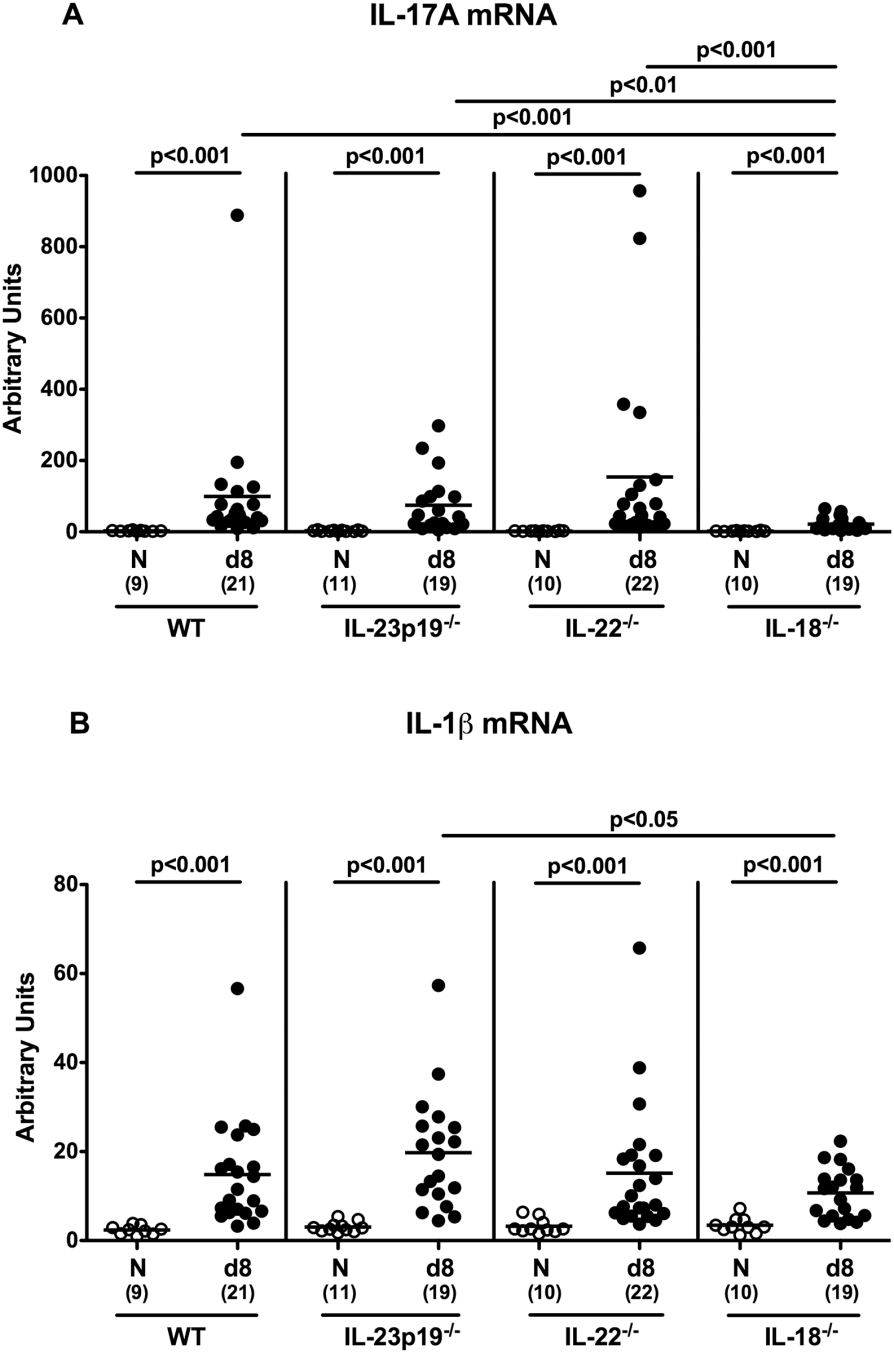
Colonic IL-17A and IL-1β mRNA expression in *C*. *jejuni* strain 81–176 infected gnotobiotic mice lacking IL-23p19, IL-22 or IL-18. **(A)** IL-17A and **(B)** IL-1β mRNA levels were determined in colonic *ex vivo* biopsies at day (d) 8 (black circles) postinfection by Real Time PCR and expressed in Arbitrary Units (fold expression). Naive (N) mice served as uninfected controls (white circles). Medians (black bars), level of significance (p-value) determined by Mann-Whitney U test, and numbers of analyzed animals (in parentheses) are indicated. Data were pooled from four independent experiments.

We next assessed colonic pro-inflammatory cytokine secretion during murine *C*. *jejuni* infection. Eight days p.i., WT, IL-23p19^-/-^ and IL-22^-/-^, but not IL-18^-/-^ mice displayed increased TNF concentrations in colonic *ex vivo* biopsies (p<0.001; Fig 5A). In addition, not only colonic TNF, but also IFN-γ levels were lower in IL-18^-/-^ mice as compared to WT, IL-23p19^-/-^ and IL-22^-/-^ mice at day 8 p.i. (p<0.001, Fig 5A and 5B), whereas *C*. *jejuni* induced increases in colonic IL-6 secretion were less pronounced in IL-18^-/-^ versus IL-23p19^-/-^ mice (p<0.05; Fig 5C). Furthermore, *C*. *jejuni* infection was accompanied by up-regulated large intestinal IL-17A and IL-1β mRNA expression (p<0.001; Fig 6). Increase in colonic IL-17A mRNA, however, was less distinct in IL-18^-/-^ mice (p<0.01–0.001 versus infected WT, IL-23p19^-/-^ and IL-22^-/-^ mice; Fig 6B), whereas IL-1β was significantly less distinctly expressed in the large intestines of IL-18^-/-^ as compared to IL-23p19^-/-^ mice at day 8 p.i. (p<0.05; Fig 6B). Taken together, IL-18 mediates *C*. *jejuni* induced pro-inflammatory cytokine responses in gnotobiotic mice.

### Colonic IL-23p19, IL-22 and IL-18 mRNA levels in *C*. *jejuni* infected gnotobiotic mice

**Fig 7:**
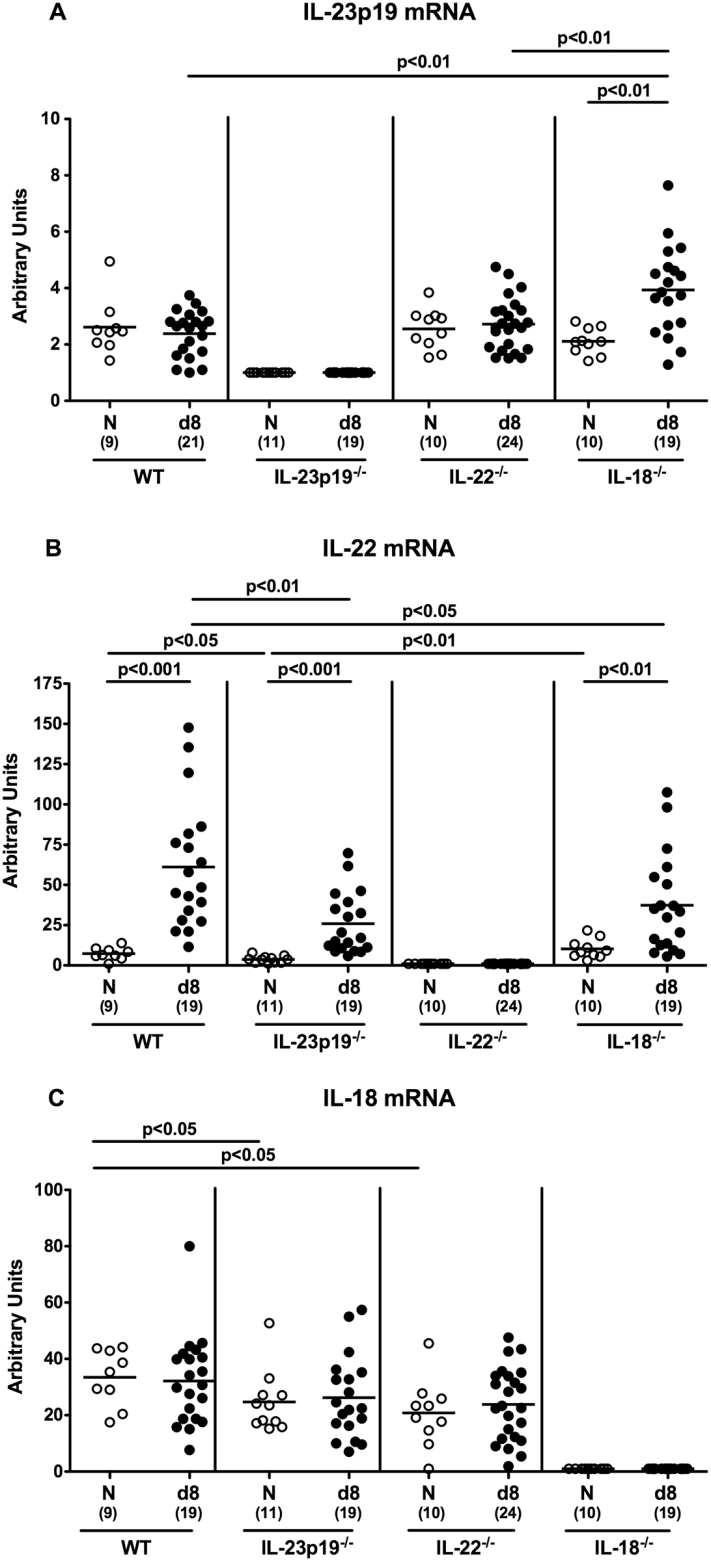
IL-23p19, IL-22, and IL-18 mRNA expression in colonic *ex vivo* biopsies derived from *C*. *jejuni* strain 81–176 infected gnotobiotic mice lacking IL-23p19, IL-22 or IL-18. **(A)** IL-23p19, **(B)** IL-22, and **(C)** IL-18 mRNA levels were determined in colonic *ex vivo* biopsies at day (d) 8 postinfection (black circles) by Real Time PCR and expressed in Arbitrary Units (fold expression). Naive (N) mice served as uninfected controls (white circles). Medians (black bars), level of significance (p-value) determined by Mann-Whitney U test, and numbers of analyzed animals (in parentheses) are indicated. Data were pooled from four independent experiments.

We next assessed whether colonic IL-23p19, IL-22 and IL-18 mRNA were differentially expressed in gnotobiotic IL-23p19^-/-^, IL-22^-/-^ and IL-18^-/-^ mice upon *C*. *jejuni* infection. As expected, IL-23p19, IL-22 or IL-18 mRNA could not be detected in colonic *ex vivo* biopsies taken from respective gene-deficient mice (Fig 7). Upon *C*. *jejuni* infection, however, IL-23p19 expression was upregulated in IL-18^-/-^ mice only and, hence, mRNA levels were higher as compared to infected mice of the remaining genotypes (p<0.01; Fig 7A). Interestingly, IL-22 mRNA levels were lower in uninfected IL-23p19^-/-^ as compared to WT and IL-18^-/-^ mice (p<0.05 and p<0.01, respectively; Fig 7B). Upon infection, colonic IL-22 mRNA expression was up-regulated, but mRNA levels were lower in IL-23p19^-/-^ and IL-18^-/-^ than WT mice at day 8 p.i. (p<0.01 and p<0.05, respectively; Fig 7B). Basal IL-18 mRNA levels, however, were slightly lower in uninfected IL-23p19^-/-^ and IL-22^-/-^ as compared to WT mice (p<0.05; Fig 7C). Remarkably, *C*. *jejuni* infection did not result in increased colonic IL-18 mRNA expression in mice of all genotypes (not significant; Fig 7C).

### Pathogenic translocation and systemic pro-inflammatory responses in *C*. *jejuni* infected gnotobiotic mice lacking IL-23p19, IL-22 or IL-18

We furthermore assessed potential systemic inflammatory responses upon *C*. *jejuni* infection. To address this, we investigated whether viable pathogens had translocated from the intestinal tract to extra-intestinal compartments including spleen, liver, kidney and cardiac blood upon peroral infection. By direct plating, *C*. *jejuni* could be isolated from spleens of only single or few WT, IL-23p19^-/-^, IL-22^-/-^ and IL-18^-/-^ mice (namely 10.0%, 11.1%, 8.3% and 18.2%, respectively) at day 8 p.i. (S2A Fig). Whereas no *C*. *jejuni* could be cultured from livers of WT and IL-23p19^-/-^ mice, 16.7% and 18.2% of livers derived from infected IL-22^-/-^ and IL-18^-/-^ mice, respectively, were pathogen-positive (S2B Fig). Moreover, 30.0%, 22.2%, 16.7% and 9.1% of WT, IL-23p19^-/-^, IL-22^-/-^ and IL-18^-/-^ mice, respectively, harbored viable *C*. *jejuni* in their kidneys (S2C Fig), whereas no pathogenic translocation into cardiac blood could be observed at all at day 8 p.i. by direct plating (S2D Fig).

**Fig 8:**
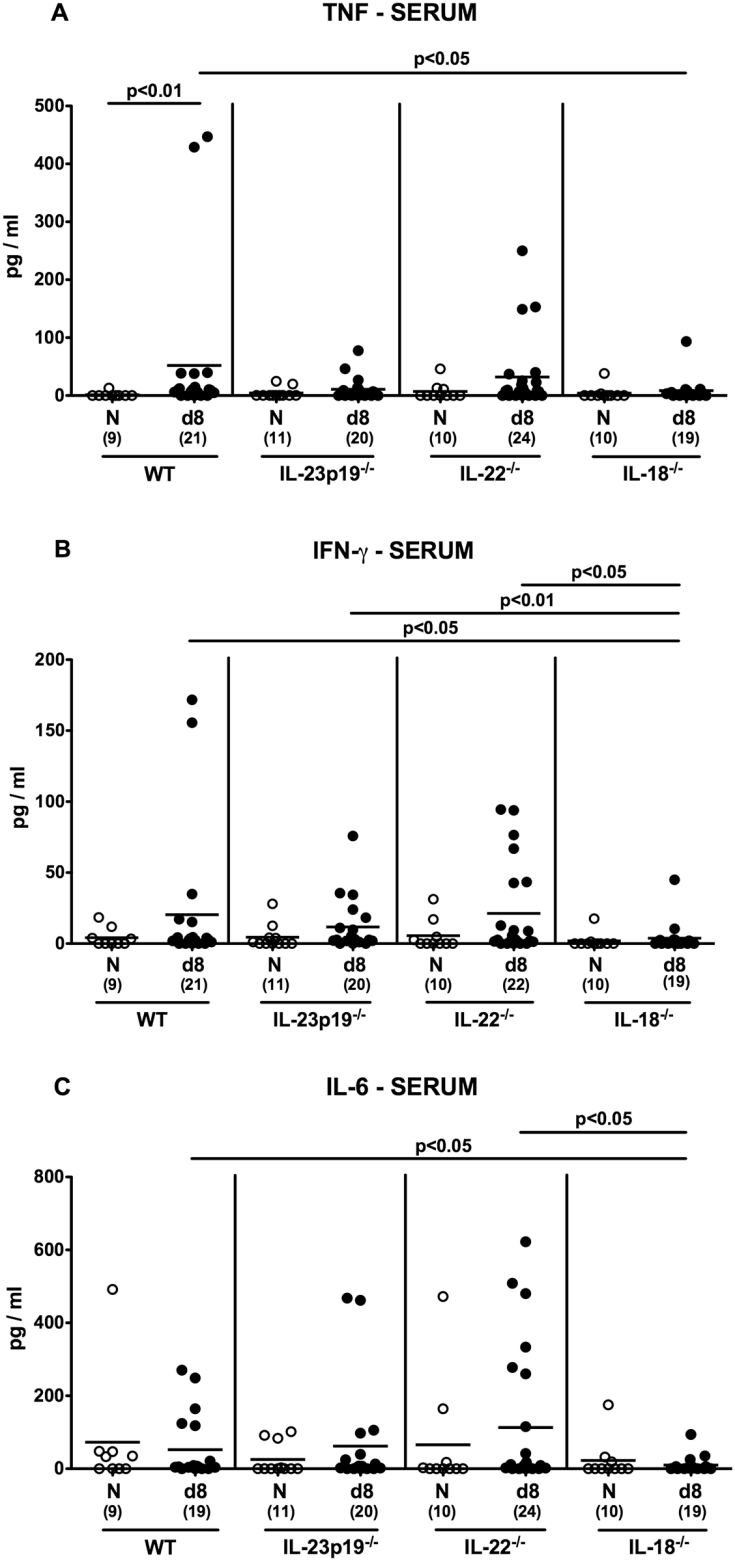
Systemic pro-inflammatory cytokine secretion in *C*. *jejuni* strain 81–176 infected gnotobiotic mice lacking IL-23p19, IL-22 or IL-18. **(A)** TNF, **(B)** IFN-γ, and **(C)** IL-6 concentrations were determined in serum samples taken from gnotobiotic wildtype (WT), IL-23p19^-/-^, IL-22^-/-^ and IL-18^-/-^ mice at day (d) 8 (black circles) postinfection. Naive (N) mice served as uninfected controls (white circles). Medians (black bars), level of significance (p-value) determined by Mann-Whitney U test, and numbers of analyzed animals (in parentheses) are indicated. Data were pooled from four independent experiments.

We further measured pro-inflammatory cytokines in serum samples taken at day 8 p.i. in order to survey systemic *C*. *jejuni* induced inflammatory responses. Remarkably, despite absence of viable pathogens in the blood stream, TNF concentrations were elevated in sera of *C*. *jejuni* infected WT (p<0.01), but not in infected mice deficient for IL-23p19, IL-22 or IL-18, as compared to respective uninfected controls (Fig 8A). Furthermore, TNF, IFN-γ, and IL-6 serum concentrations were lower in IL-18^-/-^ as compared to WT mice at day 8 p.i. (p<0.05; Fig 8). Hence, IL-18 does not only mediate local, but also systemic immune responses in *C*. *jejuni* infected gnotobiotic mice.

## Discussion

The IL-23 / IL-22 / IL-18 axis is essentially involved in host defence and in mediating and regulating inflammatory immune responses upon parasitic and bacterial infection [12–15, 23]. In the present study we shedded further spotlights onto the actors of this orchestrated interplay between *C*. *jejuni* and host immunity and applied the gnotobiotic mice infection model.

Expectedly, the physiological colonization resistance could be completely abrogated in gnotobiotic IL-23p19^-/-^, IL-22^-/-^ and IL-18^-/-^ mice upon depletion of the commensal intestinal microbiota and subsequently, irrespective of the genotype of mice, stable pathogenic colonization of the intestinal tract at comparable and high loads be warranted. In line with our previous studies [11, 17], infected mice were not clinically compromized and did not exert typical symptoms of campylobacteriosis such as wasting or bloody diarrhea. However, IL-18 was essentially involved in mediating macroscopic sequelae of *C*. *jejuni* infection, given that the large intestinal lengths significantly shortened until day 8 p.i., except for IL-18^-/-^ mice. The better macroscopic outcome in infected IL-18^-/-^ mice was also true on microscopic level, since infected IL-18^-/-^ mice displayed less abundant colonic epithelial apoptosis, but, conversely, higher numbers of proliferating cells counteracting potential infection-induced cell damage as compared to WT controls. Furthermore, IL-18 mediated adaptive immune and pro-inflammatory responses upon *C*. *jejuni* infection, given that colonic numbers of B lymphocytes and regulatory T cells were lower in infected IL-18^-/-^ as compared to WT mice. The fact that IL-18 derived from dendritic cells upon bacterial stimulation directly acts on T cells in order to facilitate conversion to FOXP3+ regulatory T cells [31] might hence explain the less pronounced increase in FOXP3+ cell numbers within the large intestinal mucosa and lamina propria of *C*. *jejuni* infected IL-18^-/-^ as compared to WT mice.

To date, however, solid data regarding the role of IL-18 in *C*. *jejuni*-host interaction, are scarce. Several *in vitro* studies demonstrated an upregulated IL-18 gene expression upon *C*. *jejuni* infection [32–34]. Moreover, genes encoding IL-23 and IL-18, but not IL-22, were regulated in differentiated macrophages following *C*. *concisus* infection as shown by transcriptomic and proteomic analyses [35].

Our *in vivo* data presented here are well in line with our previous murine infection studies applying a different gram-negative bacterial species, namely *Arcobacter butzleri* sharing taxonomic relationship with *C*. *jejuni*. As for *C*. *jejuni* [17], IL-18, but also IL-22 were up-regulated in the large intestines of *A*. *butzleri* infected mice [36, 37]. As shown by us [16, 17] and others [38], the Th-17 cytokines IL-17A, IL-22 and IFN-γ increased in the intestines of *C*. *jejuni* infected mice. Colonic expression of this cytokine triad, however, was less pronounced in gnotobiotic IL-18^-/-^ mice at day 8 p.i. as shown in the present study. Furthemore, *C*. *jejuni* induced large intestinal TNF secretion increased less distinctly in either gene-deficent as compared to WT mice. Remarkably, eventhough viable pathogens could only sporadically be isolated from extra-intestinal tissue sites, but not at all from the blood stream, elevated TNF serum levels could be observed in infected WT, but not IL-18^-/-^ gnotobiotic mice. Hence, IL-18 does not only mediate local (i.e. intestinal), but also systemic immune responses upon murine *C*. *jejuni* infection.

Our present results are at least in part supported by our very recent investigations applying a different *C*. *jejuni* infection model, namely conventionally colonized infant mice that were perorally infected immediately after weaning (i.e. by the age of three weeks) [16]. Six days following *C*. *jejuni* strain 81–176 infection, colonic numbers of both innate and adaptive immune cell subsets such as neutrophils, T and B lymphocytes were lower in IL-23p19^-/-^, IL-22^-/-^ and IL-18^-/-^ as compared to WT infant mice, whereas large intestinal Treg numbers were lower in infected IL-23p19^-/-^ and IL-22^-/-^ versus WT infant control animals [39]. In the present study, however, gnotobiotic IL-22^-/-^ mice exhibited even higher T lymphocyte and Treg numbers in their colon at day 8 p.i. Remarkably, at day 6 p.i., colonic secretion of pro-inflammatory cytokines including TNF, IFN-γ, IL-6 and MCP-1 was less pronounced in infant IL-18^-/-^ as compared to WT control mice despite stable intestinal colonization of the former only [39]. Furthermore, like in the present study, colonic IL-17A mRNA levels were lower in IL-18^-/-^ as compared to WT infant mice at day 6 p.i. [40].

Cytokines belonging to the IL-23 / IL-22 / IL-18 axis appear in fact to be differentially expressed in an orchestrated manner. Upon *C*. *jejuni* infection of gnotobiotic mice, colonic IL-23p19 expression was upregulated in IL-18^-/-^ mice only, whereas IL-22 mRNA levels were lower in uninfected and infected IL-23p19^-/-^ as well as infected IL-18^-/-^ as compared to respective wildtype control mice. These results are partly supported by our very recent infection studies in mice haboring a conventional intestinal microbiota, given that colonic IL-22 mRNA were down-regulated in infected conventional IL-23p19^-/-^ mice and vice versa (i.e. colonic IL-23p19 mRNA down-regulated in infected conventional IL-22^-/-^ mice) (Heimesaat et al., Gut Pathogens, *in press*). Notably, IL-18 mRNA was downregulated in both, uninfected and *C*. *jejuni* infected conventional IL-22^-/-^ mice, but not the other way round (Heimesaat et al., Gut Pathogens, *in press*). Differences in respective expression patterns might be explained by the different infection models including absence / presence of the complex commensal intestinal microbiota given that the interplay between pathogen and host immune system will be essentially influenced by abundance of a complex commensal microbiota.

**In conclusion,** we here show for the first time that IL-18 is essentially involved in mediating *C*. *jejuni* infection in the gnotobiotic mice model. Further studies should elucidate the underlying regulatory mechanisms orchestrating pathogen-host interactions in more detail.

## Supporting Information

S1 Fig Kinetic survey of fecal blood in perorally infected gnotobiotic mice lacking IL-23p19, IL-22 or IL-18. Gnotobiotic wildtype (WT), IL-23p19^-/-^, IL-22^-/-^ and IL-18^-/-^ mice were generated by broad-spectrum antibiotic treatment and perorally infected with *C*. *jejuni* strain 81–176 by gavage at day 0 and day 1. Abundance of fecal blood was surveyed at (A) day 1, (B) day 5, and (C) day 8 postinfection (p.i.) applying a standardized haemoccult score. Naive (N) mice served as uninfected controls. Numbers of mice with a fecal blood-positive result out of the total number of analyzed animals are given in parentheses and means (black bars) are indicated. Data were pooled from four independent experiments.
(TIFF)

S2 Fig Extraintestinal translocation of viable intestinal *C*. *jejuni* strain 81–176 in perorally infected gnotobiotic mice lacking IL-23p19, IL-22 or IL-18. Pathogenic translocation to extraintestinal compartments was assessed by determining *C*. *jejuni* strain 81–176 loads (colony forming units (CFU) per gram) in **(A)** spleen, **(B)** liver, **(C)** kidney, and **(D)** cardiac blood at day 8 (black circles) postinfection by culture. Numbers of mice harboring the pathogen out of the total number of analyzed animals are given in parentheses and medians (black bars) are indicated. Data were pooled from three independent experiments.
(TIFF)
