# Supplementary figures and images for: Interleukin-18 Mediates Immune Responses to Campylobacter jejuni Infection in Gnotobiotic Mice
Source: PLoS One. 2016 Jun 20;11(6):e0158020. doi: 10.1371/journal.pone.0158020 (PMC4913948; doi:10.1371/journal.pone.0158020)

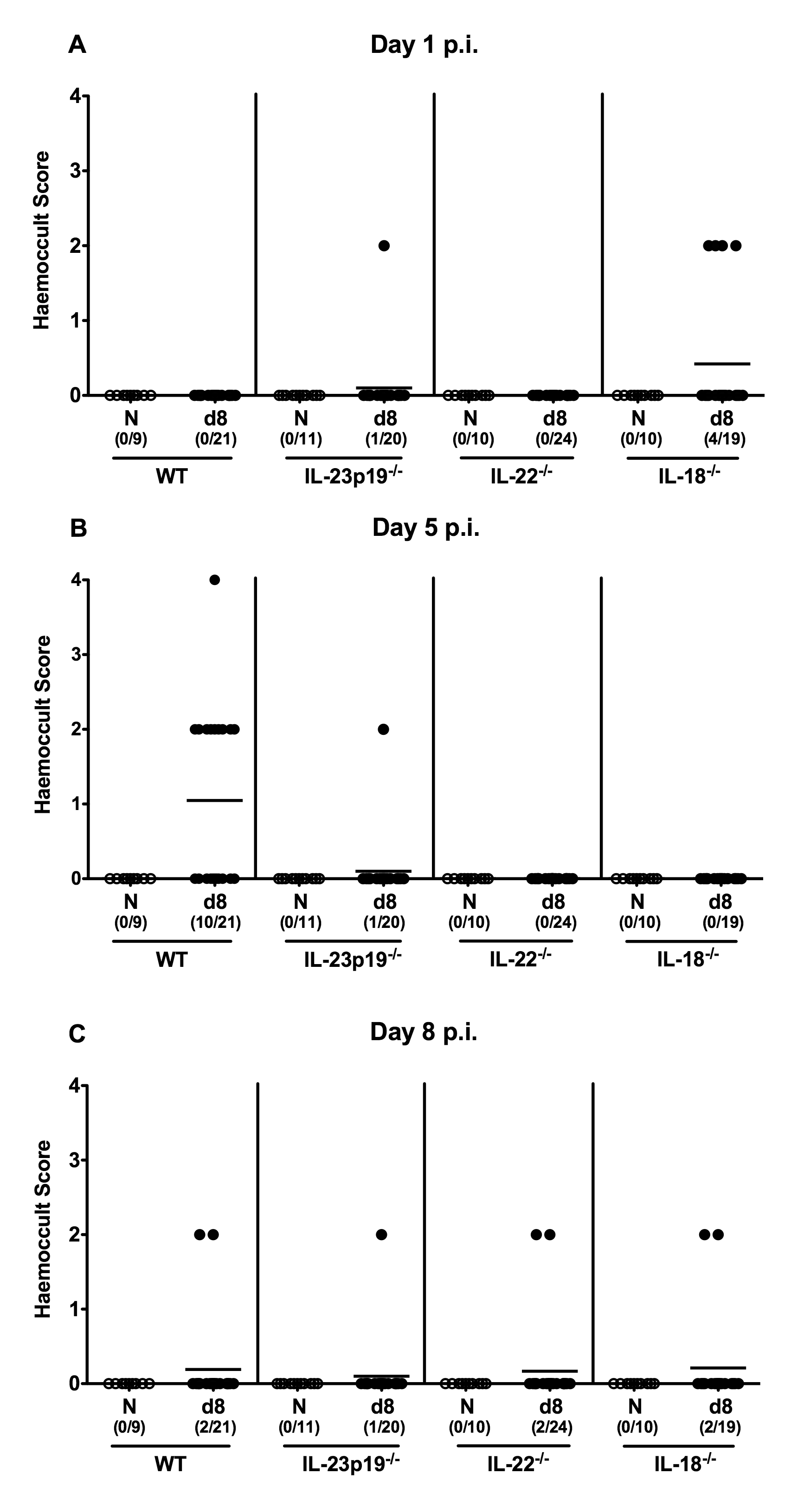

Supplement: S1 Fig — Gnotobiotic wildtype (WT), IL-23p19-/-, IL-22-/- and IL-18-/- mice were generated by broad-spectrum antibiotic treatment and perorally infected with C. jejuni strain 81–176 by gavage at day 0 and day 1. Abundance of fecal blood was surveyed at (A) day 1, (B) day 5, and (C) day 8 postinfection (p.i.) applying a standardized haemoccult score. Naive (N) mice served as uninfected controls. Numbers of mice with a fecal blood-positive result out of the total number of analyzed animals are given in parentheses and means (black bars) are indicated. Data were pooled from four independent experiments. (TIFF) [file pone.0158020.s001.tiff]

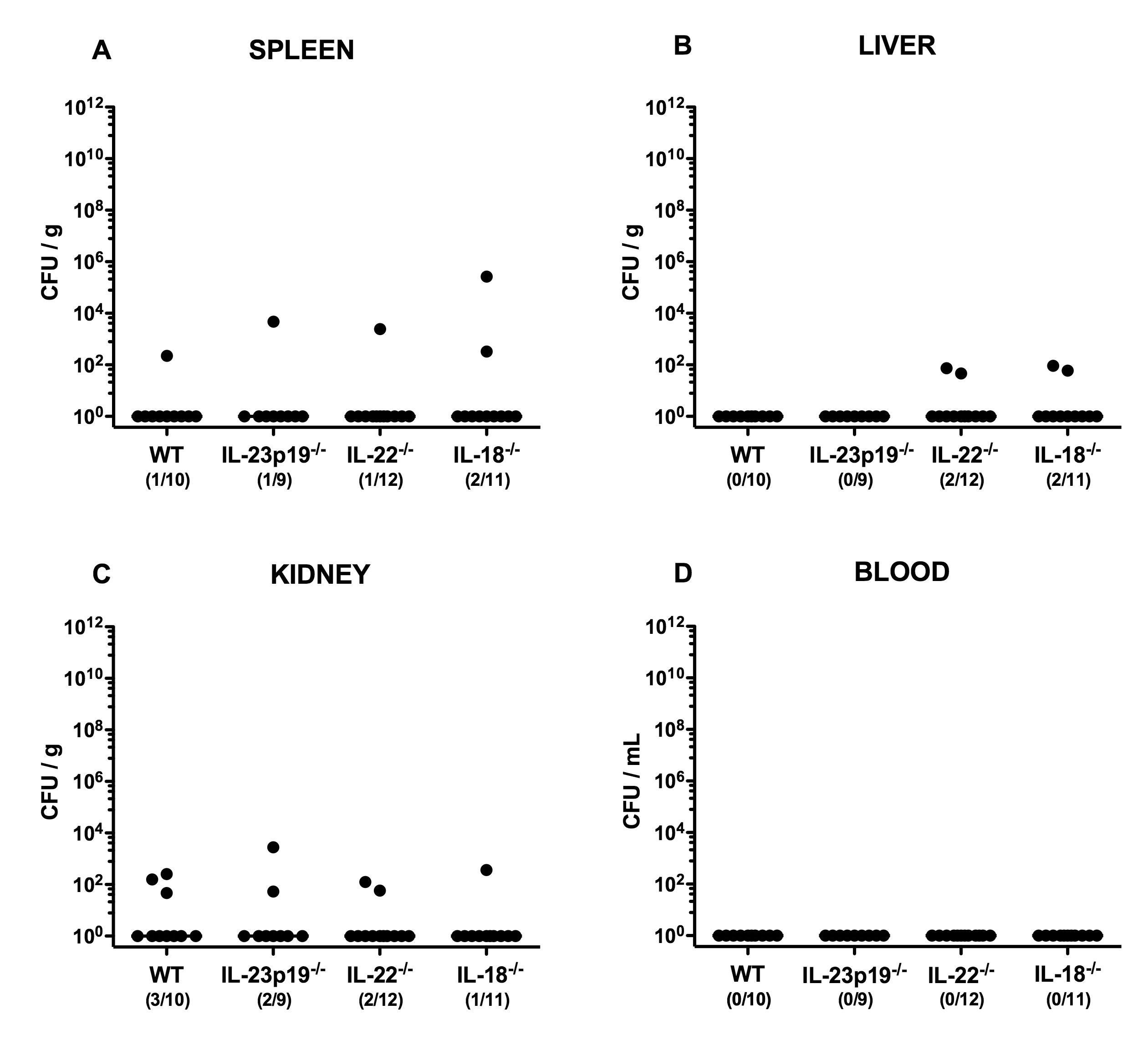

Supplement: S2 Fig — Pathogenic translocation to extraintestinal compartments was assessed by determining C. jejuni strain 81–176 loads (colony forming units (CFU) per gram) in (A) spleen, (B) liver, (C) kidney, and (D) cardiac blood at day 8 (black circles) postinfection by culture. Numbers of mice harboring the pathogen out of the total number of analyzed animals are given in parentheses and medians (black bars) are indicated. Data were pooled from three independent experiments. (TIFF) [file pone.0158020.s002.tiff]
